# Validation of an automated system for at-slaughter assessment of footpad dermatitis and hock burn in broiler chickens

**DOI:** 10.1016/j.psj.2026.106968

**Published:** 2026-04-17

**Authors:** N. van Staaveren, K. van Langeveld, J. Schulte-Landwehr, J. Mulder, P. Galliot, P. Créach, P. Sztandarski, J. Marchewka, B. Forkman, N. Van Noten, F.A.M. Tuyttens, M.F. Giersberg, M.W.E. Manet, T.B. Rodenburg

**Affiliations:** a Department of Population Health Sciences, Faculty of Veterinary Medicine, Utrecht University, 3584 CL Utrecht, the Netherlands; b Department of Animal Biosciences, Ontario Agricultural College, University of Guelph, Guelph, Ontario, N1G 2W1, Canada; c Animal Sciences Unit, Flanders Research Institute for Agriculture, Fisheries and Food (ILVO), Merelbeke-Melle, Belgium; d CLK GmbH, Altenberge D-48341, Germany; e Plukon Food Group, Wezep, the Netherlands; f Institut Technique de l'aviculture - ITAVI, Ploufragan 22440, France; g Institute of Genetics and Animal Biotechnology of the Polish Academy of Sciences, Jastrzębiec, 05-552, Magdalenka, Poland; h Department of Veterinary and Animal Sciences, University of Copenhagen, Frederiksberg, Denmark; i Department of Veterinary and Biosciences, Faculty of Veterinary Medicine, Ghent University, Merelbeke-Melle, Belgium

**Keywords:** Abattoir, Animal welfare, Broilers, Pododermatitis, Sensors

## Abstract

The automated assessment of footpad dermatitis and hock burn via camera systems at the slaughterline could allow continuous monitoring of these important welfare indicators in broiler chickens. This study aimed to compare the performance of a camera system (ChickenCheck software, CLK GmbH) against three human assessors. Footpad dermatitis (4 point scale based on relative size of the lesions compared to the footpad) and hock burn (3 point scale based on absolute size of the lesions) were scored on both the left and right foot and hock according to severity from images captured by the camera system. A total of 50 images (100 feet and hocks) were used in the training dataset used to train the human assessors and 500 images (1,000 feet and hocks) were used in the validation dataset to determine measures of agreement and reliability for the discrete scoring system. Additionally, 100 images were used for lesion annotation (200 feet and hock) to determine the agreement between the camera system and the human assessors in capturing the total area affected by lesions. Despite showing substantial to good intra-observer reliability (kappa: 0.78-0.98) and moderate to substantial inter-observer reliability (kappa: 0.46-0.66) during training, the human assessors showed fair to moderate agreement (kappa: 0.33-0.47) for footpad dermatitis and hock burn when the dataset included a larger number of images with greater variation in lesion severity and appearance. However, the human assessors showed moderate to substantial agreement with the scores assigned by the camera system (0.60-0.70). More importantly, human assessors and camera system are highly correlated when it comes to indicating the size of lesions on the footpad (R^2^: 0.93) and hock burn (R^2^: 0.97), suggesting that lesions are correctly annotated by the camera system. However, the small but systematic differences found in the size of the affected area of footpad dermatitis (+2.47%) and hock burn (-0.38 cm^2^) by the camera system compared to the human assessors need to be addressed to further improve the performance of the automated system.

## Introduction

Footpad dermatitis and hock burns are important animal-based indicators of welfare in broiler chickens. Footpad dermatitis and hock burn can cause pain and discomfort to the birds and can lead to reduced mobility (EFSA AHAW Panel (EFSA Panel on Animal Health and Welfare), 2023). While several factors influence the occurrence of footpad dermatitis and hock burn, poor litter quality is considered a main cause (Hocking and Veldkamp, 2019). Both conditions are forms of contact dermatitis, whereby the footpad and toes, or the caudal part of the hock joint, are affected by inflammation and necrotic lesions (Bessei, 2006; Shepherd and Fairchild, 2010; EFSA AHAW Panel (EFSA Panel on Animal Health and Welfare) et al., 2023). Methods with varying scoring scales, associated thresholds for different gradations, and definitions with variable levels of detail describing the (relative) size, severity, and/or depth of the lesions are available for the evaluation of footpad dermatitis and hock burn (e.g. Ekstrand et al., 1998; Welfare Quality, 2009; Ask, 2010; Michel et al., 2012; Heitmann et al., 2018; Piller et al., 2020; Louton et al., 2020; Souza et al., 2024). Most of these scoring systems are based on visual assessment of the size of the lesion, and to some extent higher visual scores are associated with higher microscopic scores and depth of inflammation, thus mirroring the histological processes observed when footpad dermatitis or hock burn occurs (Heitmann et al., 2018; Piller et al., 2020; Louton et al., 2020).

Information on footpad dermatitis and hock burn can be collected on farm and in the slaughterhouse, with the latter having some distinct advantages. As nearly all broiler chickens end up at the slaughterhouse at the end of their lives, the central position of the slaughterhouse in the broiler production chain allows the collection of large amounts of data on broiler chicken flocks in a relatively short time. Animal-based measures collected at the slaughterhouse can function as an iceberg indicator of on-farm welfare to assess the cumulative welfare of broiler chickens (Tuyttens et al., 2025). In case of high percentages of birds with lesions, management of future broiler chicken flocks on the same farm can be improved to reduce their incidence. In the EU, the Council Directive 2007/43/EC is a main driver for EU Member States to monitor animal-based measures such as footpad dermatitis and hock burn at slaughter (EFSA, 2021). This directive includes monitoring of flock mortality and footpad dermatitis at slaughter. While data on footpad dermatitis and hock burn is typically collected through visual inspection by competent authorities or food business operators, this is labour intensive and open to low inter- and intra-observer reliability. Therefore, there is also increasing interest in automated methods to capture this data. Several camera systems for automated monitoring of footpad dermatitis and hock burn at slaughter are already commercially available and used in some countries e.g., The Netherlands or Germany (EFSA, 2021; Voogt et al., 2023). The need to validate such technologies is well recognized, but validation is generally limited (Tuyttens et al., 2022; Voogt et al., 2023). Validation involves both sensor output validation and confirming the relevance of the output to animal welfare (H.-L. Ko, Autonomous University of Barcelona, Barcelona, Spain, personal communication). The relevance of footpad dermatitis and hock burn for the welfare of broiler chickens is well established (EFSA AHAW Panel (EFSA Panel on Animal Health and Welfare) et al., 2023), but few studies have validated the sensor output as automated systems are developed for footpad dermatitis (Vanderhasselt et al., 2013; van Harn and de Jong, 2017; Louton et al., 2022a) and hock burn in broiler chickens (Louton et al., 2022b). Automated systems generally consist of a camera that captures images of feet or hocks, and software that determines (relative) size of the discolouration that represents the lesions observed when footpad dermatitis or hock burn is present. This is then used to categorize the severity of footpad dermatitis and hock burn, similar to the scoring systems used by human assessors that assess the size/colour of lesions. As such, first studies focused on determining the agreement between the sensor and human assessors and determining thresholds for the different scoring categories (Vanderhasselt et al., 2013; van Harn and de Jong, 2017; Louton et al., 2022a; b). While these studies have shown generally promising results in agreement between sensors and human assessors, the authors also highlighted limitations due to variation in methods (e.g., flock vs individual bird level, different scoring scales used by sensor and human rater, scoring of live birds, feet/hocks at slaughter or digital images) and emphasized the need to evaluate and update software as necessary in the specific context (Vanderhasselt et al., 2013; van Harn and de Jong, 2017; Louton et al., 2022a; b). Within different slaughterhouses conditions may vary (e.g., different light conditions, line speeds, camera lenses, camera distance, type of birds processed) which requires fine-tuning of the camera system and software.

Therefore, the aim of this study was to evaluate the performance of a computer vision technology for automated scoring of footpad dermatitis and hock burn recently installed in two commercial broiler chicken slaughterhouses as part of a larger project, as recent guidelines suggest to repeat the validation procedure if a sensor is applied in a different context (H.-L. Ko, Autonomous University of Barcelona, Barcelona, Spain, personal communication). To determine whether the sensor output measured the intended animal-based measure (i.e., footpad dermatitis and hock burn), we compared the system’s performance to that of human assessors in several steps. Firstly, we assessed the intra- and inter-observer reliability in ordinal scoring of footpad dermatitis and hock burn by human assessors who then served as a silver standard to later evaluate the performance of the automated system. Secondly, we determined the correspondence between the output of the sensor (camera score) and discrete scoring by the human assessors. Finally, we determined the correspondence between the sensors and human assessors in terms of the size of the area affected by footpad dermatitis (relative size) or hock burn (absolute size) as this is the underlying output on which the camera score is assigned.

## Materials and methods

This study was part of the aWISH (‘Animal Welfare Indictaors at the SlaughterHouse’) project (https://www.awish-project.eu/), which aims to develop methods for the automated assessment of animal welfare at the slaughterhouse. Two commercial broiler chicken slaughterhouses (slaughterhouse DUC, France, line speed: 12,000 birds per h, slaughterhouse Plukon Sieradz Sp.z o.o, Poland, line speed: 15,000 birds per h) with camera systems equipped with ChickenCheck software of CLK GmbH (Altenberge, Germany) were used to collect images of footpads and hocks against a blue background plate. The camera systems consist of one camera (1.3 MP color camera (IDS Imaging, Obersulm, Germany) with two light units, each equipped with LED lights flashing at 74 Hz. The camera systems were installed immediately after plucking/defeathering for the hocks, and after the point where the feet are separated from the broiler carcass so that only the feet are present in the slaughterline for the images of the footpads. As this study relied on existing data from commercially slaughtered broiler chickens no ethical approval was required. No information was available on the origin of the broiler chickens (e.g., flock, batch, genotype, weight/age) for this dataset.

### Scoring systems for footpad dermatitis and hock burns

**Fig. 1:**
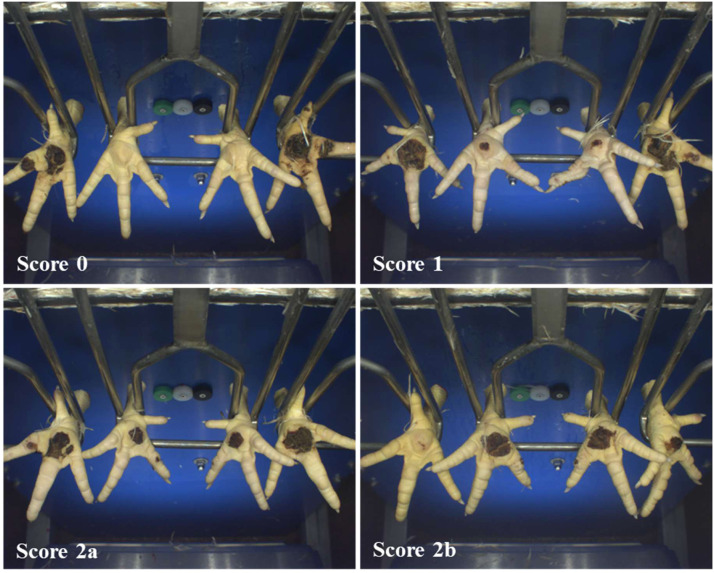
Example images of footpad scores (0, 1, 2a, 2b) as captured by the camera-system in the slaughterhouse. The pair of feet in the middle shackle represent the score. The green outline indicates the footpad reference area and the red outline indicates the lesion area. Images provided by CLK GmbH.

**Fig. 2:**
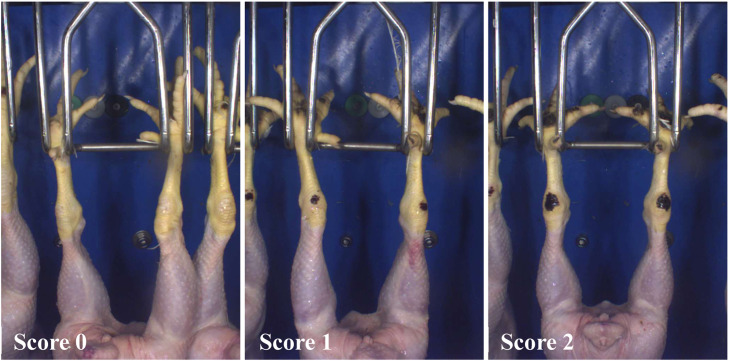
Example images of hock burn scores (0, 1, 2) as captured by the camera-system in the slaughterhouse. Images provided by CLK GmbH.

The scoring system of footpad dermatitis followed the QS Guideline Diagnostic Data in Poultry Slaughtering (QS, 2025), as this system is used as a standard in multiple EU countries as part of quality assurance schemes. This system used a four point scale for footpad dermatitis (described below). Lesions on the footpad were evaluated in comparison to the reference images provided; no written definitions were provided for the different scores. Scores (0, 1, 2a, 2b) were assigned according to the relative surface of the footpad covered by lesions (hereafter relative size), with a higher score indicating a larger area affected by lesions (Fig. 1). Lesions on the toes were not included in the assessment. For hock burn, the scoring system was adapted from the Dutch IKB Kip assurance scheme, which assesses hock burn as present or absent (PLUIMNED, 2021). An additional score category was added in the current study so that a 0-2 scoring system was used based on the absolute size of the lesions on the hock, where a higher score indicates a larger lesion (Fig. 2).

The camera system assessed footpad dermatitis and hock burn based on colour differences compared with the surrounding area to determine the size (pixels) of the lesion. Both the left and right foot and hock were assessed separately. In the case of footpad dermatitis, the camera system determined the total area of the footpad as a reference area through morphological operations on the biggest inner circle of the segmented foot region. This was used to finally calculate the relative area (%) affected by footpad lesions (absolute lesion size in pixels divided by the absolute footpad size in pixels). For hocks, the absolute size (cm^2^) of the lesions was calculated using a conversion factor based on the palne where the hock burn lesions are located to go from pixels to cm^2^. The (relative) size of the lesions was used to assign a particular score category for each indicator based on confidential cut-offs.

All images were uploaded to the Computer Vision Annotation Tool (CVAT) (CVAT.ai Corporation, 2024) for annotating and allowing the assessors to independently perform the required tasks to evaluate footpad dermatitis and hock burn.

### Initial scoring of footpad dermatitis and hock burn (training)

The three assessors participating in the study all had prior experience (4-10 years) with scoring footpad dermatitis or hock burn, although their backgrounds differed. The first two assessors had experience through the Welfare Quality scoring system for laying hens and broilers (assessor 1 on-farm, assessor 2 in research flocks), whereas assessor 3 had experience scoring footpad dermatitis in broilers and rabbits.

The training process consisted of multiple steps starting with an initial meeting to discuss the scoring systems for footpad dermatitis and hock burn, including the provided guidelines with reference images covering both clear and borderline cases of the different score categories. During this initial meeting, approx. 50 images were presented and discussed among the project team to calibrate the assessors. Additionally, instructions for use of the Computer Vision Annotation Tool (CVAT) were provided. Following this, the three experienced assessors proceeded to individually evaluate the images of feet and hocks of an initial 50 broilers (*initial training dataset*). The initial dataset was selected by CLK from previously scored images by the camera system in such a way that they gave a clear visual of the feet and hocks (from one slaughterhouse) and included relatively equal representation of all possible scores (Supplementary Table 1). Importantly, this dataset predominantly contained clear and representative examples of each score category, with limited inclusion of borderline or ambiguous cases. The purpose of this was to facilitate calibration and alignment between assessors. Each assessor scored the left and right foot and hock in separate tasks, giving a final 100 scores for footpad dermatitis and 100 scores for hock burn. The exercise was repeated later with the same images in a randomly different order to assess the intra-observer reliability of each assessor. Following this training, the assessors discussed the results of their scoring in relation to each other and the camera system before continuing with a larger dataset for the final scoring of footpad dermatitis and hock burn.

### Final scoring of footpad dermatitis and hock burn (validation)

A larger dataset of 500 broilers (*final scoring dataset*) was constructed similarly to the initial dataset with images with equal representation of both slaughterhouses. This dataset also included relatively equal representation of all possible scores, but with more variation in severity (including a wider range of lesion sizes within each score categories and thus more borderline cases, better reflecting real-world conditions) compared to the initial dataset (Supplementary Table 1). Each rater scored the left and right foot and hock in separate tasks, similar as during the training, giving a final 1,000 scores for both footpad dermatitis and hock burn.

### Annotation of areas affected by footpad dermatitis and hock burn

In a final step, assessors manually drew the area(s) affected by footpad dermatitis and hock burn on each image of the left and right foot and hock in a dataset of 100 broilers (*lesion area dataset*), resulting in the annotation of 200 feet and 200 hocks. Images in the dataset came from both slaughterhouses and contained an equal number of all scores greater than 0 (score 0 was excluded as no annotation would be needed) to ensure all lesion sizes were represented. In the CVAT system, assessors could draw a polyline as a connected sequence of line segments around the affected area(s), and this was compared to the area outlined by the algorithm within the camera system on the same images in terms of relative size for footpad dermatitis or absolute size for hock burn. This allowed us to check if the assessors and the automated camera system identified the same total area of the lesion.

### Data analysis

All measurements were analyzed using SAS V9.4 (SAS Inst. Inc., Cary, NC). Multiple measures of agreement and reliability were assessed for the scoring of footpad dermatitis and hock burn in the initial training and final scoring dataset. Intra-observer reliability was evaluated via simple kappa statistics using only the initial training dataset. Both the initial and final dataset were used to evaluate the inter-observer reliability between the three human assessors. The number of assessors agreeing on each image was determined, and the percentage of images where all assessors, 2 assessors or none of the assessors agreed on the score was calculated. Inter-observer reliability was further assessed by calculating estimates and tests of agreement between multiple assessors using the %MAGREE macro (v3.8, SAS Institute Inc., Cary, NC, USA). Kappa statistics were calculated to assess the level of agreement corrected for chance. In particular, the exact and weighted kappa statistics were estimated. Linear weighting was used to quantify the relative difference between score categories by different assessors and to attribute partial credit depending on the extent of disagreement between assessors using Fleiss–Cohen weights (Watson and Petrie, 2010). A smaller penalty is given when assessors disagree to a smaller extent (e.g., 1-point difference) compared to when they disagree to a larger extent (e.g., 2 or 3-point difference). When the kappa value is positive, the observed agreement exceeds chance agreement, with a higher value indicating a stronger agreement. Classifications of kappa values suggested by Landis and Koch (1977) and presented in Petrie and Watson (2013) were used for interpretation, where *κ* ≤ 0.20 is ‘poor’, 0.21 ≤ *κ* ≤ 0.40 is ‘fair’, 0.41 ≤ *κ* ≤ 0.60 is ‘moderate’, 0.61 ≤ *κ* ≤ 0.80 is ‘substantial’, and *κ* > 0.80 is ‘good’. It should be noted that these thresholds are arbitrary and are only used to ease interpretation; generally speaking, values closer to 1 indicate a higher level of agreement. To test the agreement of the rater’s ranking of the images, the Kendall’s W coefficient of concordance was estimated. Additionally, Gwet’s weighted agreement coefficient (AC_2_) for ordinal data was estimated to assess agreement on each score category while considering both assessors and images as sampled rather than fixed to assess agreement of the entire populations of assessors and images. Values closer to 1 indicate a higher level of agreement for both Kendall’s *W* and Gwet’s agreement coefficient.

To assess how scoring by individual assessors compared to that of the camera system, first, the median of the scores of the three assessors was calculated to reflect the silver standard. Following this, kappa statistics (simple kappa as well as linear and quadratic kappa) were estimated to compare the median score of the three assessors and the score of the camera system.

Finally, the agreement between human assessors and the camera system in terms of the area of the footpad (relative area, %) or hock (absolute area, cm^2^) affected by lesions in the annotation exercise was assessed. As a first step, a linear regression was used (PROC REG) with the mean area affected by lesions determined by the three human assessors as the explanatory variable for the area affected by lesions as assigned by the camera system. As a second step, a generalized linear mixed model was used to evaluate the effect of the rating system (camera, average of the three human assessors), side (left or right), and their interaction on the annotated area affected by lesions. A Tukey–Kramer adjustment was used to account for multiple comparisons. Assumptions of normally distributed residuals and homogeneity of variance were examined graphically. Statistical significance was considered at P < 0.05 and tendencies are reported at 0.05 ≤ P ≤ 0.1.

## Results

### Intra- and inter-observer reliability between assessors for footpad dermatitis and hock burn

The intra-observer reliability for footpad dermatitis and hock burn was substantial to good for each rater during initial training (Table 1). The scores assigned by the assessors and the camera system for all images in the initial training and final validation dataset are presented in Supplementary Table 1.

**Table 1:** Agreement within three assessors when scoring footpad dermatitis (score 0, 1, 2a, 2b) and hock burn (score 0, 1, 2) in the initial training dataset (initial; 50 images for a final 100 scores) of left and right feet and hock. The 95% confidence interval for kappa is given in brackets.

	Footpad dermatitis	Hock burn
Rater 1	0.88 (0.80-0.95)	0.95 (0.90-1.00)
Rater 2	0.88 (0.80-0.95)	0.98 (0.95-1.00)
Rater 3	0.88 (0.80-0.95)	0.78 (0.68-0.89)

During the initial training there were many cases for both footpad dermatitis and hock burns where all three assessors agreed on the score and no cases where all three assessors disagreed (Table 2). This was especially the case for hock burn and was also reflected in the substantial kappa values. In comparison, the kappa value for footpad dermatitis was moderate when considering exact agreement and substantial when linear weighing for partial agreement was applied. For both footpad dermatitis and hock burn, Kendall’s W was high (≥0.95), indicating near perfect agreement for the ranking of the images, and the Gwet’s agreement coefficient overall was close to 1, showing good agreement as well. Upon closer evaluation, the Gwet’s agreement coefficients were lower for the more intermediate scores, i.e., score 1 and 2a for footpad dermatitis and score 1 for hock burns.

**Table 2:** Agreement between three assessors when scoring footpad dermatitis (score 0, 1, 2a, 2b) and hock burn (score 0, 1, 2) in the initial training dataset (initial; 50 images for a final 100 scores) and final validation dataset (final; 500 images for a final 1,000 scores) of left and right feet and hock. The 95% confidence interval for kappa, linear weighted kappa, Kendall’s W, and Gwet’s AC_2_ coefficients are given in brackets.

	Footpad dermatitis		Hock burn	
	Initial	Final	Initial	Final
*Percentage agreement (%)*				
All 3 assessors agree	80.0%	34.3%	97.0%	82.1%
2 assessors agree	20.0%	58.0%	3.0%	17.9%
No assessors agree	0%	7.7%	0%	0%
Kappa	0.46 (0.41-0.50)	0.33 (0.30-0.36)	0.66 (0.62-0.70)	0.47 (0.32-0.62)
Linear weighted kappa	0.65 (0.61-0.69)	0.52 (0.48-0.56)	0.75 (0.72-0.78)	0.59 (0.45-0.73)
Kendall’s W	0.95 (0.93-0.98)	0.90 (0.89-0.91)	0.99 (0.98-1.00)	0.93 (0.92-0.94)
*Gwet’s AC_2_*				
Overall	0.90 (0.84-0.95)	0.61 (0.21-1.00)	0.98 (0.93-1.00)	0.88 (0.80-0.95)
Score 0	0.98 (0.93-1.00)	0.95 (0.91-0.99)	0.98 (0.92-1.00)	0.93 (0.89-0.98)
Score 1	0.88 (0.78-0.98)	0.67 (0.38-0.95)	0.97 (0.91-1.00)	0.84 (0.75-0.93)
Score 2 (hock)	NA^1^	NA^1^	1.00	0.92 (0.85-0.99)
Score 2a (footpad)	0.87 (0.80-0.93)	0.56 (0.22-0.91)	NA^1^	NA^1^
Score 2b (footpad)	0.96 (0.91-1.00)	0.75 (0.43-1.00)	NA^1^	NA^1^

1 Score 2a and 2b only exist for footpad dermatitis, while score 2 only exists for hock burn.

In the final scoring dataset, the level of agreement decreased between the three assessors, particularly for footpad dermatitis (Table 2). While for hock burn, there still were no cases where all three assessors disagreed on the scores, this was observed in nearly 8% of the cases for footpad dermatitis. Moreover, only in approx. 34% of the cases did all three assessors agree on the score for footpad dermatitis, and the value for the kappa statistic similarly dropped to fair agreement as opposed to the moderate agreement seen in the initial dataset (and to moderate as opposed to substantial agreement when considering linear weighting). Similarly, the kappa values for hock burn in the final dataset were moderate as opposed to substantial in the initial dataset. The sources of disagreement again related mainly to the intermediate scores as shown by the lower Gwet AC_2_ coefficients in comparison to the extreme scores, while there was general agreement on the overall ranking of the images as indicated by the high Kendall’s W (Table 2).

### Agreement between human assessors and the camera system for scores of footpad dermatitis and hock burn

The median scores for footpad dermatitis and hock burn of the three human assessors were compared to those given by the algorithm from the camera system. Measures of agreement between assessors and the camera were generally better during the initial training than during the final scoring (Table 3). Based on the median of the three assessors as the silver standard, there was moderate agreement between the assessors and the camera system for hock burn (kappa: 0.60) and substantial agreement for footpad dermatitis (kappa: 0.70) in the final dataset.

**Table 3:** Agreement between each individual assessors as well as the median of the three assessors and the camera system when scoring footpad dermatitis (score 0, 1, 2a, 2b) and hock burn (score 0, 1, 2) in the initial training dataset (initial; 50 images for a final 100 scores) and final validation dataset (final; 500 images for a final 1,000 scores) of left and right feet and hock. The 95% confidence interval for kappa, linear weighted kappa and quadratic weighted kappa are given in brackets.

	Rater 1 vs Camera		Rater 2 vs Camera		Rater 3 vs Camera		Rater median vs Camera	
	Initial	Final	Initial	Final	Initial	Final	Initial	Final
*Footpad dermatitis*								
Exact agreement (%)	82.0%	51.7%	94.0%	78.5%	87.0%	61.9%	91.0%	77.7%
Simple kappa	0.76 (0.65-0.86)	0.36 (0.32-0.40)	0.92 (0.86-0.98)	0.71 (0.68-0.75)	0.82 (0.73-0.91)	0.49 (0.45-0.53)	0.88 (0.80-0.95)	0.70 (0.67-0.74)
Linear weighted kappa	0.84 (0.77-0.91)	0.60 (0.57-0.62)	0.95 (0.90-0.99)	0.81 (0.78-0.83)	0.88 (0.82-0.94)	0.65 (0.71-0.77)	0.92 (0.87-0.97)	0.80 (0.77-0.82)
Quadratic weighted kappa	0.91 (0.87-0.95)	0.78 (0.75-0.80)	0.97 (0.95-0.99)	0.89 (0.87-0.91)	0.93 (0.90-0.97)	0.79 (0.77-0.81)	0.96 (0.93-0.98)	0.88 (0.87-0.90)
*Hock burn*								
Exact agreement (%)	94.0%	80.9%	97.0%	71.5%	97.0%	69.8%	97.0%	73.4%
Simple kappa	0.91 (0.84-0.98)	0.71 (0.67-0.74)	0.95 (0.90-1.00)	0.58 (0.54-0.61)	0.95 (0.90-1.00)	0.56 (0.52-0.59)	0.95 (0.90-1.00)	0.60 (0.57-0.64)
Linear weighted kappa	0.93 (0.87-0.98)	0.78 (0.75-0.81)	0.96 (0.92-1.00)	0.66 (0.63-0.70)	0.96 (0.92-1.00)	0.65 (0.61-0.68)	0.96 (0.92-1.00)	0.69 (0.66-0.72)
Quadratic weighted kappa	0.95 (0.91-0.99)	0.85 (0.83-0.87)	0.97 (0.94-1.00)	0.76 (0.74-0.79)	0.97 (0.94-1.00)	0.75 (0.72-0.78)	0.97 (0.94-1.00)	0.78 (0.76-0.81)

### Comparison of lesion annotation for footpad dermatitis and hock burn by individual assessors and the camera system

**Fig. 3:**
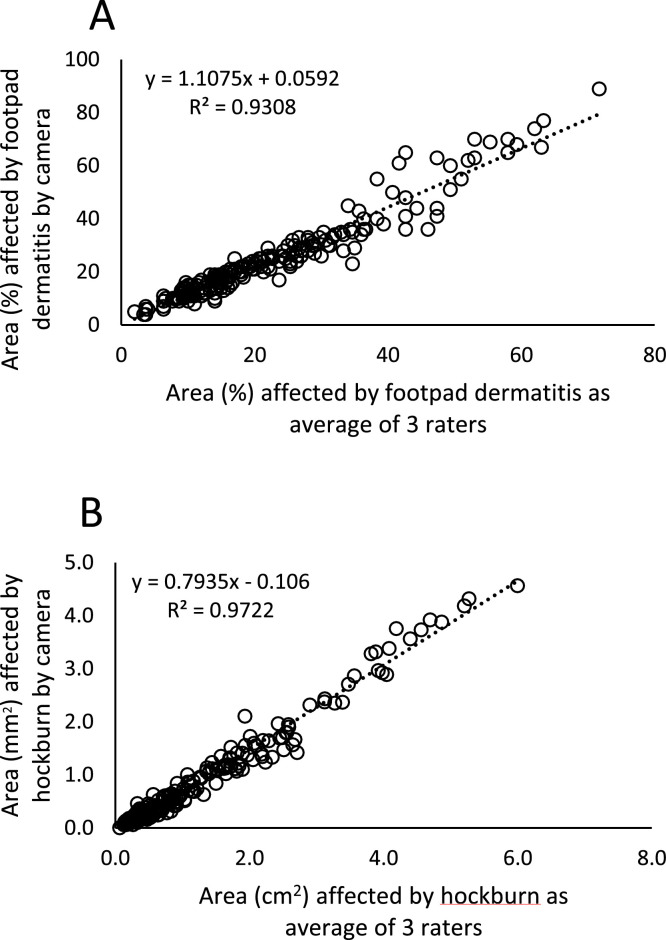
Linear regression for A) footpad dermatitis and B) hock burn comparing the overall area affected as assessed by the camera system and the average of the three individual assessors. The area of footpad dermatitis was assessed as a relative size and expressed as percentage (%) of the entire footpad. Hock burn was expressed as absolute size (cm^2^).

The relative and absolute size of the area affected by footpad dermatitis and hock burn, respectively, as determined by the camera system and individual assessors are presented in Table 4. In contrast to the discrete scoring system, correlations between the three assessors’ annotation of the area affected by lesions were high (r > 0.95) for both footpad dermatitis and hock burn (Supplementary Table 2). Moreover, the correlations for the size of the lesions based on the average of the three human assessors and the camera system were high for both footpad dermatitis (Fig. 3A) and hock burns (Fig. 3B). For each +1% increase in area affected by footpad dermatitis as assessed by human raters there was a + 1.1% increase in area affected as assessed by the camera system, while for each +1 cm^2^ increase in area affected by hock burn there was a + 0.79 cm^2^ increase in area affected as assessed by the camera system.

**Table 4:** Descriptive statistics for the annotation of the relative size affected by footpad dermatitis (%) and absolute size affected by hock burn (cm^2^) as determined by the camera system and individual assessors. The annotation dataset contained images of 100 broilers giving a final 200 feet and hocks for annotation.

	Mean	SD	Min	Max	95% CLM^1^
Footpad dermatitis (%)					
Camera	24.8	16.1	4	89	22.6-27.1
Rater 1	22.8	14.6	2	75	20.7-24.8
Rater 2	21.5	12.8	2	61	19.8-23.3
Rater 3	22.8	15.1	2	79	20.7-24.9
Hock burn (cm^2^)					
Camera	0.93	0.96	0	4.57	0.79-1.06
Rater 1	1.29	1.20	0	5.98	1.13-1.46
Rater 2	1.32	1.20	0.10	6.00	1.16-1.49
Rater 3	1.29	1.20	0	6.01	1.13-1.46

1 95% confidence limit of the mean.

### Differences between camera system and assessors in lesion annotation for footpad dermatitis and hock burns

Differences between the camera system and the average of three human assessors existed for the relative size of footpad dermatitis (F_3,322.1_ = 7.47, P = 0.0066) and absolute size of hock burn (F_1,297_ = 154.64, P < 0.001) (Table 5). The relative size of footpad dermatitis was larger according to the camera system (+2.47%, t_322.1_ = 2.73, P = 0.0066), while for hock burns the camera system indicated a smaller area compared to the three human assessors (−0.38 cm^2^, t_297_ = −12.44, P < 0.001).

**Table 5:** Least square means of the relative size of footpad dermatitis (%) and absolute size of hock burn (cm^2^) according to rating system (the camera and average of the three individual assessors), side (left, right) and their interaction. Different superscript letters indicate significant differences (P < 0.05) within each lesion. The annotation dataset contained images of 100 broilers giving a final 200 feet and hocks for annotation.

	Footpad dermatitis (%)	Hock burn (cm^2^)
*Rating system*	*F_3,322.1_=7.47 P=0.0066*	*F_1,297_=154.64, P<0.001*
Camera	25.41 ± 1.71 ^a^	0.93 ± 0.11 ^a^
Human	22.94 ± 1.71 ^b^	1.30 ± 0.11 ^b^
*Side*	*F_1,322.1_=0.93, P=0.3345*	*F_1,297_=25.48, P<0.001*
Left	24.61 ± 1.71	1.04 ± 0.11 ^a^
Right	23.74 ± 1.71	1.19 ± 0.11 ^b^
*Interaction*	*F_1,322.1_=0.05, P=0.8232*	*F_1,297_=0.16, P=0.6896*
Camera – Left	25.74 ± 1.82	0.86 ± 0.11
Camera – Right	25.07 ± 1.82	1.00 ± 0.11
Human – Left	23.48 ± 1.82	1.22 ± 0.11
Human – Right	22.41 ± 1.82	1.39 ± 0.11

Finally, regardless of whether the annotation was done by the camera system or the human assessors, the area annotated on the left hock was smaller (1.03 ± 0.11 cm^2^) than the area on the right hock (1.19 ± 0.11 cm^2^, F_1,297_ = 25.48, P < 0.001). No such difference was observed between the left and right side for the annotation of the relative area affected by footpad dermatitis (left: 24.61 ± 1.71% vs right: 23.74 ± 1.71%, F_1,322.1_ = 0.93, P = 0.3345).

## Discussion

This study evaluated the performance of an automated camera system to assess footpad dermatitis and hock burns in broiler chickens at the slaughterline. This validation focused on the output of the ChickenCheck system for the assessment of footpad dermatitis and hock burns in a multi-step approach by comparing its performance against three human assessors. Validation of the welfare relevance of these indicators is another aspect of validation (H.-L. Ko, Autonomous University of Barcelona, Barcelona, Spain, personal communication) but was out of the scope of the current study.

### Agreement within and between individual assessors (intra-observer and inter-observer reliability)

The human assessors were generally experienced with assessing footpad dermatitis and hock burn in poultry. During initial training on the scoring systems used, all three assessors showed high intra-observer reliability (range: 0.78-0.98). This is comparable to other reported values for intra-observer reliability (e.g., 0.88 for footpad dermatitis in turkeys (Stracke et al., 2022)) and suggests all assessors had a consistent internal standard. In terms of inter-observer reliability between all three assessors, the exact kappa values were lower, but still moderate for footpad dermatitis (0.46) and substantial for hock burns (0.66). In contrast, Piller et al. (2020) reported kappa values between 0.87 to 0.89 for footpad dermatitis, and Louton et al. (2020) reported kappa values between 0.75 to 0.87 for hock burns, with both studies using a 0 to 4 scoring scale. However, these studies compare specific pairs of assessors and thus the reported kappa value depends on the specific pair. In the current study, we used an approach to assess the agreement between all assessors simultaneously (i.e., in this case all three assessors had to agree), as also used by Vanderhout et al. (2022), which could partly explain the lower kappa values in the current study. Pairwise comparisons between the three assessors in the current study indeed did find similarly high kappa values for footpad dermatitis (0.79-0.85) and hock burn (0.86-0.95) during initial training (Supplementary Table 3).

The finding that the assessors had a higher agreement during training than during the final scoring exercise can be due to the final dataset containing more images with more variation in lesion severity (e.g., meaning also more borderline cases) and occasionally lower quality images due to practical conditions in the slaughterhouses (e.g., less clear images due to foggy conditions). These may have complicated scoring by the human assessors who subsequently agreed less with each other. This would be especially the case for the intermediate scores which are generally more difficult to agree on than the extremes (Decina et al., 2019; Vanderhout et al., 2022). Additionally, the assessors scored based on a visual scale from example images without detailed definitions. Heitmann et al. (2018) encountered similar difficulties in agreement between assessors for intermediate scores when terms such as ‘small’, ‘medium’, or ‘deep’ were left open to interpretation. This may also explain why agreement for footpad dermatitis was more difficult to obtain than for hock burns in the current study. Footpad dermatitis had firstly more scoring categories, and secondly required a relative assessment based on size of the lesion compared to the overall size of the footpad. The difficulty humans have in accurately assessing (relative) size has been pointed out previously (Heitmann et al., 2018; Toppel et al., 2019). This is also reflected in the finding that the different score categories that assessors assigned were often overlapping in terms of the size of the area as determined by the camera system while the camera system provided a clearer distinction between the score categories as it functioned based on specific thresholds (Supplementary Fig. 1). Interestingly, Stracke et al. (2022) showed a somewhat higher agreement between assessors who scored ‘live’ feet as opposed to images (Krippendorff’s alpha of 0.82 vs 0.77), which they attributed to the lack of spatial perception of the foot when scoring from images.

### Agreement between individual assessors and the camera system

Based on the median of the three assessors as the silver standard, the individual assessors generally agreed well with the camera during initial training in terms of scores for footpad dermatitis (kappa: 0.88) and hock burns (kappa: 0.95). As these values and the intra- and inter-observer reliability between assessors were sufficient during training, assessors proceeded to score the final scoring dataset.

When presented with the final scoring dataset, there was moderate agreement between the assessors and the camera system for hock burn (kappa: 0.60) and substantial agreement for footpad dermatitis (kappa: 0.70). Likely, a similar explanation as for the reduced agreement between the assessors when assessing the final scoring dataset is at play here, however, it is interesting to note that it is dependent on the specific rater and lesion scored as suggested by the larger variability in kappa values ranging from 0.36 to 0.71 for footpad dermatitis and 0.56 to 0.71 for hock burn. Promisingly, only very few cases were due to discrepancies of more than one score category difference between a rater and the camera system. The majority of differences came from a one-point difference in the score attribution i.e., only 26 out of 1,000 cases for footpad dermatitis and 5 out of 1,000 cases for hock burns showed a difference of more than 1 score category between a rater and the camera system.

Previous studies also found variable correlations (range: 0.54 - 0.97) between human assessors and automated systems for footpad dermatitis (Vanderhasselt et al., 2013; van Harn and de Jong, 2017), with correlations generally being higher when looking at flock level scores or once incorrect or incomplete automated assessments were removed. Louton et al. (2022a; b) similarly validated the performance of the ChickenCheck software to assign specific scores based on specific thresholds of the area affected by footpad dermatitis and hock burn. While they used different scoring systems (0-4 scoring scale) compared to the current study, they also highlighted that adaptations to the software led to improvements for the performance measures of the camera system when assigning the scores for footpad dermatitis and, to lesser extent, for hock burn. Similarly, following the results of the study and discussion of the current study, changes were made in the software to adjust the determination of the footpad reference area on the right foot by the camera system. This also emphasises the importance of output validation of automated systems in specific contexts (H.-L. Ko, Autonomous University of Barcelona, Barcelona, Spain, personal communication).

The output of the camera system used in the current study is the size of the lesions based on which the discrete scores are assigned. As an additional step in the validation process, the three assessors therefore annotated the area affected by lesions for footpad dermatitis and hock burn. In this case, the annotation performed by the three assessors agreed well with the camera system (Supplementary Fig. 2). The variance in lesion size observed by the camera system was largely explained by the average lesion size as indicated by the three assessors (R^2^ ≥0.93). This output validation is an important step and suggests that the camera system is accurately identifying the footpad dermatitis and hock burn lesions, despite potentially minor differences observed in overall size between the camera system and assessors. The camera system estimated the area of footpad dermatitis as being larger and the area of hock burns as being smaller than the human assessors did. One explanation could be that the camera system may include lesions of footpad dermatitis on/between the toes that human assessors would no longer consider to be on the footpad. In contrast, for hock burn, human assessors could be quicker to deem something a lesion than the camera system. Previously, Louton et al. (2022a) found a low sensitivity of the automated camera system in identifying footpad dermatitis of a low score (i.e., single superficial lesion or several cumulated superficial lesions or deep lesion ≤0.5 cm). While this concerns a different lesion (i.e. footpad dermatitis instead of hock burn), it does suggest that minor lesions do not get picked up by the system. However, it should be acknowledged that for both footpad dermatitis and hock burn the differences between the camera system and the assessors were small, and it could be questioned how biologically significant these differences are. Nevertheless, this requires further investigation and fine-tuning to avoid systematic differences between human and automated system when such an automated system would be used for monitoring purposes. All in all, it is likely that the camera system is better at translating the sizes of the lesions to the different scoring categories for footpad dermatitis and hock burn in contrast to the assessors who show more variability (Supplementary Fig. 2). It could be argued that providing the actual size of the lesion may provide a more accurate reflection. In practice, the scoring categories are used during inspections to calculate average flock scores based on the number of birds with the specific scores. In the current study, it was not possible to ascertain which images came from specific flocks, and so no flock level scores could be calculated. However, previous work by Vanderhasselt et al. (2013) and van Harn and de Jong (2017) has suggested that human and automated assessments of footpad dermatitis generally correlate on a flock level. A large advantage of the automated camera system is that it can score all birds within a flock and monitor continuously, compared to a human rater who is limited in the amount of data that can be collected due to the line speed.

### Limitations of the study

The human assessors in this study scoring from images were considered as a silver standard. A gold standard where trained assessors could assess the feet and hocks under the best possible circumstances (e.g., with real feet and hocks, with no time pressure, perfect lighting, etc.) would be ideal (H.-L. Ko, Autonomous University of Barcelona, Barcelona, Spain, personal communication), but was not feasible in the current study. However, Stracke et al. (2022) reported better agreement between an automated camera system and human assessors for footpad dermatitis in turkeys when the human assessors scored from images as compared to ‘live’ scoring. When scoring from images, circumstances are different than when scoring ‘live’; assessors are under less pressure as they do not have to deal with the slaughterhouse environment (e.g., high line speeds, noise), but nuances may also be lost, and image quality plays a role. While some images were foggy, overall image quality in the current study was considered to be good by the human assessors. However, it is also possible that the human and the camera make the same mistake based on the images. It is therefore important to make sure the angle and quality of the photos allow a full view of the lesions. Alternatively, a study compairing live scoring with less distractions (time pressure, movement) to scoring from images, both by humans, would give more insight on the reliability of scoring from images, and therefore of automated scoring.

Considering that the agreement between the automated camera system and the individual assessors was variable when assessing the final scoring dataset, it appears that the automated system is more consistent in assigning the ordinal scores (Supplementary Fig. 1). However, it should be acknowledged that certain aspects cannot be assessed based on images and are thus not accounted for in the current algorithm, such as the depth of lesions, which is an important aspect of footpad dermatitis. However, lesion depth is also difficult to assess visually by human assessors (and would require palpation of the feet), and the size of the lesion has been suggested to function as a proxy for lesion depth (Heitmann et al., 2018).

Finally, it should be noted that it was not possible to evaluate the repeatability of the camera system itself, e.g., by passing the same feet/hocks through the slaughterline multiple times. This would be an interesting aspect to consider as it may have revealed differences due to the way in which the feet and hocks are angled as they pass the camera system.

## Conclusion

The automated assessment of footpad dermatitis and hock burn could allow continuous monitoring of these important welfare indicators in broiler chickens. Validation of automated systems for monitoring animal welfare is limited and requires that sensor output is validated against a gold (or silver) standard. We aimed to fill this knowledge gap by comparing the performance of the automated ChickenCheck software camera system, which assigned scores for footpad dermatitis and hock burn based on the (relative) size of the lesions, to the scores assigned by three human assessors who served as the silver standard. Assessors generally agreed with each other for the discrete scores of footpad dermatitis and hock burn during training; however, when the dataset became larger and more complex, there was more variability between assessors. As such, the silver standard showed moderate to substantial agreement with the discrete scores assigned by the camera system. Promisingly, the variance in lesion size observed by the camera system was largely explained by the average lesion size as indicated by the assessors (R^2^ ≥0.93) with coefficients of +1.1% for footpad dermatitis and +0.79 cm^2^ for hock burn by the automated system for each 1-unit increase in size by the assessors. The small but systematic differences found in the size of the affected area between humans and the system need to be addressed to further improve the performance of the automated system. These results suggest that the camera system is more adept at translating the (relative) size of lesions into the discrete scoring categories than humans and furthermore has the benefit of continuous large-scale monitoring of broiler chicken flocks in a uniform way.

## Declaration of competing interest

J. Schulte-Landwehr is an employee of CLK GmbH, Germany. All other authors declare no conflict of interest.

## CRediT authorship contribution statement

**N. van Staaveren:** Writing – original draft, Visualization, Methodology, Investigation, Formal analysis, Data curation, Conceptualization. **K. van Langeveld:** Writing – review & editing, Methodology, Investigation. **J. Schulte-Landwehr:** Writing – review & editing, Software, Methodology, Investigation, Data curation. **J. Mulder:** Writing – review & editing, Supervision, Resources, Methodology, Investigation. **P. Galliot:** Writing – review & editing, Validation, Methodology, Investigation. **P. Créach:** Writing – review & editing, Supervision, Investigation, Funding acquisition, Data curation. **P. Sztandarski:** Writing – review & editing, Validation, Methodology, Investigation, Data curation. **J. Marchewka:** Writing – review & editing, Supervision, Methodology, Funding acquisition, Conceptualization. **B. Forkman:** Writing – review & editing, Supervision, Methodology, Conceptualization. **N. Van Noten:** Writing – review & editing, Supervision, Project administration, Funding acquisition, Conceptualization. **F.A.M. Tuyttens:** Writing – review & editing, Supervision, Methodology, Funding acquisition, Conceptualization. **M.F. Giersberg:** Writing – review & editing, Supervision, Methodology, Conceptualization. **M.W.E. Manet:** Methodology, Validation, Writing – review & editing. **T.B. Rodenburg:** Writing – review & editing, Supervision, Project administration, Methodology, Funding acquisition, Conceptualization.

## Disclosures

J. Schulte-Landwehr is an employee of CLK GmbH, Germany. All other authors declare no conflict of interest.

## Appendix Supplementary materials

Image, application 1

Image, application 2

Image, application 3

Image, application 4

Image, application 5

## Footnotes

Supplementary material associated with this article can be found, in the online version, at doi:10.1016/j.psj.2026.106968.
