# Supplementary material for: Validation of an automated system for at-slaughter assessment of footpad dermatitis and hock burn in broiler chickens
Source: Poult Sci. 2026 Apr 17;105(7):106968. doi: 10.1016/j.psj.2026.106968 (PMC13141726; doi:10.1016/j.psj.2026.106968)
Supplement: Supplementary file 1 [file mmc1.docx]

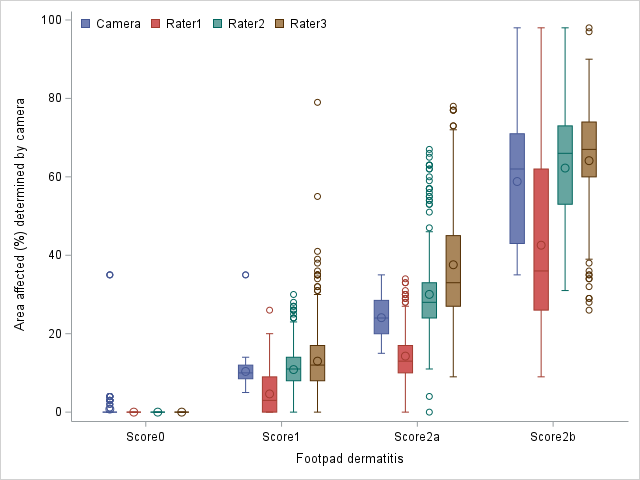

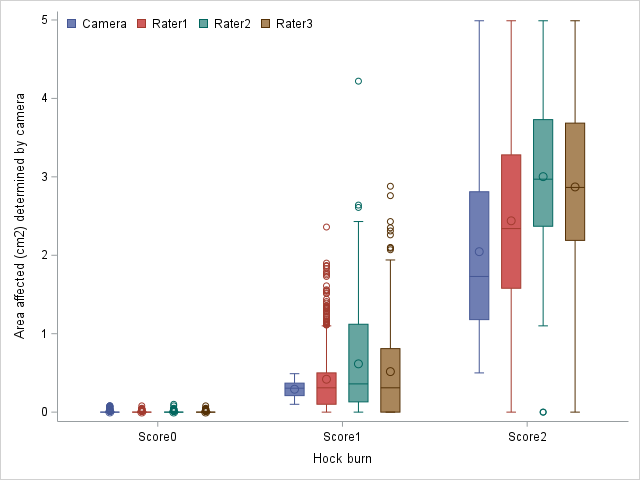


A

B

Supplementary Figure 1 Distribution of the area affected by A) footpad dermatitis and B) hock burn as determined by the camera system and the associated scores given by the different assessors in the final scoring dataset of 500 images of left and right feet and hock (final of 1,000 scores per lesion type)..
