# Supplementary material for: Validation of an automated system for at-slaughter assessment of footpad dermatitis and hock burn in broiler chickens
Source: Poult Sci. 2026 Apr 17;105(7):106968. doi: 10.1016/j.psj.2026.106968 (PMC13141726; doi:10.1016/j.psj.2026.106968)
Supplement: Supplementary file 2 [file mmc2.docx]

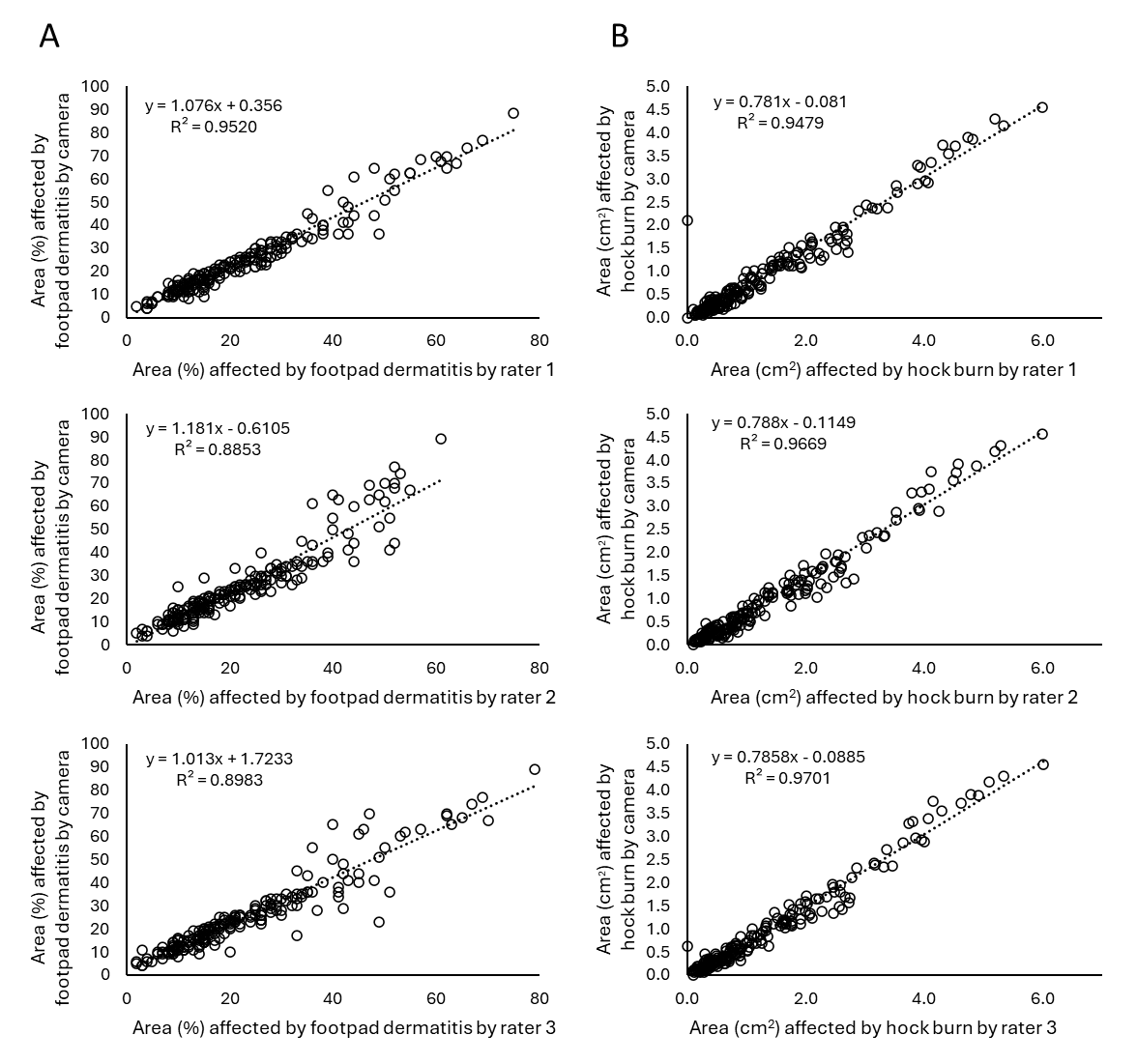


Supplementary Figure 2 Linear regression for A) footpad dermatitis and B) hock burn comparing the overall area affected as assessed by the camera system and the three different assessors. The area of footpad dermatitis was assessed as a relative size and expressed as percentage (%) of the entire footpad. Hock burn was expressed as absolute size (cm^2^).
