# Supplementary material for: Validation of an automated system for at-slaughter assessment of footpad dermatitis and hock burn in broiler chickens
Source: Poult Sci. 2026 Apr 17;105(7):106968. doi: 10.1016/j.psj.2026.106968 (PMC13141726; doi:10.1016/j.psj.2026.106968)
Supplement: Supplementary file 3 [file mmc3.docx]

Supplementary Table 1 Distribution of scores (%) assigned to the images in the initial and final dataset by the three assessors and the camera system when scoring footpad dermatitis (score 0, 1, 2a, 2b) and hock burn (score 0, 1, 2). The initial dataset contained 50 images (100 scores for left and right feet/hock) and the final dataset contained 500 images (1,000 scores for left and right feet/hock) per lesion type.

|  | Initial | | | |  | Final | | | |
| --- | --- | --- | --- | --- | --- | --- | --- | --- | --- |
|  | Rater 1 | Rater 2 | Rater 3 | Camera |  | Rater 1 | Rater 2 | Rater 3 | Camera |
| *Footpad dermatitis (%)* |  |  |  |  |  |  |  |  |  |
| Score 0 | 21.0 | 18.0 | 18.0 | 20.0 |  | 12.3 | 18.0 | 15.3 | 23.7 |
| Score 1 | 39.0 | 36.0 | 34.0 | 30.0 |  | 18.5 | 37.5 | 49.1 | 24.4 |
| Score 2a | 23.0 | 26.0 | 33.0 | 30.0 |  | 27.0 | 28.2 | 24.6 | 30.4 |
| Score 2b | 17.0 | 20.0 | 15.0 | 20.0 |  | 42.2 | 16.3 | 11.0 | 21.5 |
| *Hock burn (%)* |  |  |  |  |  |  |  |  |  |
| Score 0 | 23.0 | 20.0 | 20.0 | 21.0 |  | 40.4 | 39.2 | 35.4 | 49.7 |
| Score 1 | 38.0 | 41.0 | 41.0 | 38.0 |  | 38.2 | 47.9 | 49.8 | 19.8 |
| Score 2 | 39.0 | 39.0 | 39.0 | 41.0 |  | 21.4 | 12.9 | 14.8 | 30.5 |
