# Supplementary material for: Validation of an automated system for at-slaughter assessment of footpad dermatitis and hock burn in broiler chickens
Source: Poult Sci. 2026 Apr 17;105(7):106968. doi: 10.1016/j.psj.2026.106968 (PMC13141726; doi:10.1016/j.psj.2026.106968)
Supplement: Supplementary file 4 [file mmc4.docx]

Supplementary Table 2 Correlation between the three assessors for the area affected by footpad dermatitis and hock burn based on images of 100 broilers for 200 feet and hocks, respectively.

|  | Footpad dermatitis | Hock burn |
| --- | --- | --- |
| Rater 1 vs Rater 2 | 0.97 | 0.98 |
| Rater 1 vs Rater 3 | 0.98 | 0.98 |
| Rater 2 vs Rater 3 | 0.96 | 0.99 |
