# Supplementary material for: Validation of an automated system for at-slaughter assessment of footpad dermatitis and hock burn in broiler chickens
Source: Poult Sci. 2026 Apr 17;105(7):106968. doi: 10.1016/j.psj.2026.106968 (PMC13141726; doi:10.1016/j.psj.2026.106968)
Supplement: Supplementary file 5 [file mmc5.docx]

Supplementary Table 3 Agreement between specific pairs of assessors when scoring footpad dermatitis (score 0, 1, 2a, 2b) and hock burn (score 0, 1, 2) in the initial dataset of 50 images of left and right feet and hock (final of 100 scores per lesion type) and in the final dataset of 500 images of left and right feet and hock (final of 1,000 scores per lesion type). The 95% confidence interval for kappa, linear weighted kappa and quadratic weighted kappa are given in brackets.

|  | Rater 1 vs 2 | | Rater 1 vs 3 | | Rater 2 vs 3 | |
| --- | --- | --- | --- | --- | --- | --- |
| *Footpad dermatitis* | Initial | Final | Initial | Final | Initial | Final |
| Exact agreement (%) | 86.0% | 45.7% | 85.0% | 41.0% | 89.0% | 74.2% |
| Simple kappa | 0.8077 (0.7137-0.9017) | 0.2889  (0.2495-0.3282) | 0.7940 (0.6973-0.8907) | 0.2412  (0.2045-0.2778) | 0.8492  (0.7651-0.9333) | 0.6320  (0.5936-0.6703) |
| Linear weighted kappa | 0.8737 (0.8095-0.9379) | 0.5380  (0.5071-0.5689) | 0.8610  (0.7924-0.9296) | 0.4387  (0.4053-0.4721) | 0.8985 (0.8401-0.9568) | 0.7388  (0.7097-0.7679) |
|  |  |  |  |  |  |  |
| *Hock burn* |  |  |  |  |  |  |
| Exact agreement (%) | 97.0% | 87.3% | 97.0% | 85.6% | 100% | 91.3% |
| Simple kappa | 0.9536 (0.9021-1.000) | 0.7987  (0.7669-0.8306) | 0.9536  (0.9021-1.000) | 0.7733  (0.7396-0.8069) | 1 (1.000-1.000) | 0.8559  (0.8270-0.8848) |
| Linear weighted kappa | 0.9631 (0.9222-1.000) | 0.8344 (0.8079-0.8610) | 0.9631 (0.9222-1.000) | 0.8131  (0.7847-0.8415) | - | 0.8770  (0.7097-0.7679) |
